# Photoacoustic microscopy for studying mechano-transduction response in resistance vessels

**DOI:** 10.1016/j.pacs.2025.100713

**Published:** 2025-04-04

**Authors:** Juliana Benavides-Lara, Dianicha Santana Nunez, Mohsin Zafar, Janette Garcia Campos, Shuangping Zhao, Yulia A. Komarova, Kamran Avanaki

**Affiliations:** a Richard and Loan Hill Department of Biomedical Engineering, University of Illinois at Chicago, Chicago, IL, USA; b Department of Pharmacology and Regenerative Medicine at the University of Illinois Chicago, Chicago, IL 60607, USA; c Departments of Dermatology and Pediatrics, University of Illinois Chicago, Chicago, IL 60607, USA

**Keywords:** Photoacoustic microscopy, Hypertension, Piezo1, Vessel diameter, Blood pressure, Vasodilation

## Abstract

Cardiovascular diseases are on the rise, presenting a significant global health challenge. The development of methods enabling the detection of alterations in vascular networks is critical for the early diagnosis and treatment of cardiovascular diseases, including peripheral arterial disease, stroke, and hypertension. Here, we use photoacoustic microscopy (PAM), a non-invasive imaging technique, to monitor morphological changes within the skin vessels of chronically hypertensive mice deficient in the mechanosensitive channel Piezo1 in endothelial cells (Piezo1 EC-KO). We show that, compared to control mice (Piezo1 flox/flox), Piezo1 EC-KO mice are characterized by poorer tissue perfusion due to a vasoconstriction of resistance arterioles. We also show the effect of administration of pharmacological agents on vessel vasodilation in the skin of Piezo1-deficient mice and control mice, identifying quantitative differences between the two groups. These results advance our understanding of vascular mechanodynamics and offer potential implications for developing targeted treatments for hypertensive disorders.

## 1 Introduction

Hypertension is a major cardiovascular health problem affecting nearly half of the U.S. population. It is a key risk factor for cardiovascular diseases, including heart attack and stroke [1]. Mortality rates related to hypertension are extremely high, leading to 8.5 million deaths each year [2]. Effective management of hypertension can significantly reduce this risk; hence, it is critical to further understand minute changes in local arterial blood flow as correlated with arteriole vessel diameter, with the goal of developing novel pharmacological approaches to treat this deadly disease.

Hypertension is a chronic increase in arterial blood pressure, which creates continuous mechanical stress that damages the blood vessel walls [1]. This, in part, leads to structural changes in the blood vessels causing arterioles and capillary rarefaction and decreased tissue perfusion [3]. In healthy individuals, arterial blood pressure is finely regulated by endothelial responses to local changes in blood flow [4]. Endothelial cell sensing of increased blood pressure and fluid shear is an essential adaptive process that promotes vasodilation and a subsequent reduction in local blood pressure, thereby preventing excessive stress on the vessel walls. These responses are attributed to the function of Piezo1, a mechanosensitive non-selective ion channel expressed on the plasma membrane of endothelial cells [5], [6].

Upon activation, Piezo1 undergoes conformational changes that facilitate the influx of divalent ions, including calcium, from extracellular space into endothelial cells [7]. The resulting increase in intracellular free calcium ions triggers the calmodulin-dependent transcient activation of endothelial nitric oxide synthase (eNOS), an enzyme that produces nitric oxide (NO), a potent vasodilator[8]. NO relaxes vascular smooth muscle cells by activating guanylate cyclase, which increases cyclic guanosine monophosphate (cGMP) production and ultimately leads to an increased diameter of resistance vessels [9]. This response plays a critical role in modulating vascular resistance and local arterial blood pressure [10], [11].

Deficiency of Piezo1 in endothelial cells of mice leads to chronic hypertension due to impaired NO production and reduced vasodilation [9]. Piezo1 integrates the NO-mediated vasodilation response in the endothelium through the interaction of multiple cellular pathways. For instance, Piezo1 activation is coupled with ATP release and the consequent activation of P2Y2 receptors, which is required for shear stress-induced activation of a mechanosensitive junctional complex [12]. This complex includes Platelet Endothelial Cell Adhesion Molecule-1 (PECAM-1), Vascular Endothelial (VE)-cadherin, and Vascular Endothelial Growth Factor Receptor 2 (VEGFR2) [13]. Activation of this junctional complex, in turn, triggers AKT-dependent phosphorylation of eNOS. In contrast to the calmodulin-dependent pathway, this mechanism leads to the sustained production of NO. Concurrently, shear stress dependent activation of Piezo1 induces the production adrenomedullin, a vasodilator peptide that amplifies eNOS activity via PKA dependent phosphorylation [14]. Thus, Piezo1 activity modulates NO production and vasodilation through the integration of distinct cellular pathways.

Our recent data demonstrate that Piezo1 mechanotransduction leading to eNOS activation involves soluble adenylyl cyclase (sAC), the enzyme responsible for generating cyclic adenosine monophosphate (cAMP). This, in turn, potentiates calcium release from the inositol trisphosphate receptor 2 (IP3R2) stores [15]. The resulting secondary wave of calcium elevation is required for the activation of Akt1, which is essential for sustained NO production and endothelial cell adaptive responses to increased shear stress [16]. Overexpression of membrane-localized Akt1 restores the vasodilatory responses of resistance mesenteric arteries in endothelial-specific Piezo1 deficient mice [15], suggesting that pharmacological activation of Piezo1 downstream signaling holds potential for treating hypertension, even in the absence of Piezo1 function.

Photoacoustic imaging has become an increasingly valuable technology for brain imaging and other organs, with extensive testing in various clinical and preclinical applications [17], [18], [19], [20], [21], [22], [23], [24]. Here using photoacoustic microscopy (PAM), a non-invasive method to visualize minute changes in vessel diameter, our study shows that pharmacological activation of sAC promptly increases the vessel diameter in mice with Piezo1 deficiency in endothelial cells (Piezo1 EC-KO) and restores tissue perfusion. This study also shows that the method developed here can be utilized to screen therapeutic interventions using vessel diameter as a direct endpoint measurement.

## 2 Materials and methods

### 2.1 sLS-PAM experimental setup

**Fig. 1:**
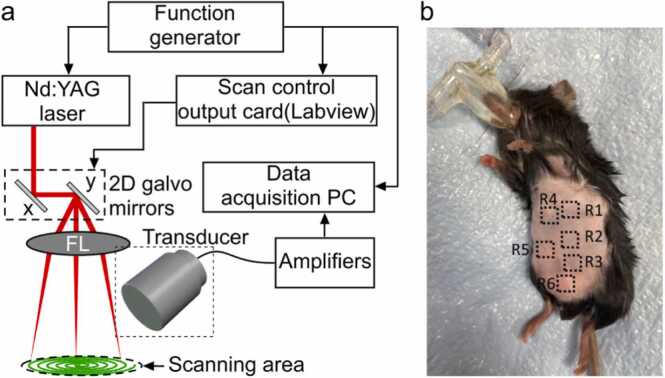
sLS-PAM system design and configuration for vessel analysis in mouse skin. (a) and positioned right beneath the thin film. Ultrasound gel was applied between the imaging target and the thin film for improved acoustic coupling. The pulse repetition rate (PRR) of the laser was 100 kHz. (b) Regions of interest.

The sLS-PAM system used a 532 nm pulsed Nd:YAG laser (VPFL-G-20, VGEN, Tel Aviv, Israel) as the optical excitation source. Collimated beams were directed onto a 2D galvanometer (GVS 202–2D, Thorlabs, Newton, NJ, USA) via two 45° mirrors (MPD019-G01, Thorlabs, Newton, NJ, USA). Sinusoidal signals of varying amplitude and phase controlled the galvanometer mirrors to create a spiral scanning pattern. These signals were generated in LabVIEW and sent to the galvanometer using an output card (BNC 2110, National Instruments, TX, USA). A 5 V peak-to-peak frame trigger signal (0.625 Hz frequency, 50 % duty cycle) was produced in LabVIEW to start data acquisition on the rising edge. Synchronization of laser irradiation, raster scanning, and frame triggering was done via a common 5 V square wave trigger (100 kHz frequency, 50 % duty cycle) from an external function generator (ATF20B, ATTEN, USA). The laser beam was focused onto the imaging object through an achromatic doublet lens (AC127–075-A, Thorlabs, Newton, NJ, USA) with a 10 cm focal length. Further details on the spiral scanning mechanism are available in references [25], [26], [27]. A hollow water tank was utilized, with its base covered by a thin, optically transparent film, Saran Wrap. The imaging target is positioned in the center of the laser scanning area, beneath the water tank. The Saran Wrap allows the laser light to pass through and reach the imaging target. The acoustic waves generated from the imaging target penetrate through the Saran wrap and travel back through water onto the transducer for detection. The schematic of the experimental is shown in Fig. 1.

A total of 160,000 points were scanned in one complete spiral frame of 1 cm diameter in 1.6 s. in the abdomen of the mouse skin. FL: focusing lens; PC: personal computer. During the scanning process, the transducer remained stationary, and its active sensing area was submerged in distilled water to ensure proper acoustic coupling. A variable gain voltage amplifier (Model 351 A, Analog Modules Inc., FL, USA) with a maximum gain of up to 80 dB was used to amplify the detected photoacoustic (PA) signals. These signals were then digitized using a 4-channel data acquisition device (CSE1642, GAGE, USA). Finally, the lateral resolution of the system is 25 µm, which is determined by the spot size of the laser on the brain.

### 2.2 Preparation of animals

Animal care and handling were performed according to an approved protocol of the University of Illinois at Chicago Animal Care Committee. Mice were housed with food and water in the UIC animal care facility in a 12 hour light and dark cycle. Piezo1 flox/flox and Piezo1 EC-KO (Piezo1 flox/flox crossed with Cdh5(PAC)-Cre^ERT2^) mice were generated, authenticated, and maintained as previously described [28]. Briefly, the Piezo1 EC-KO transgenic mice were generated by crossing Piezo1 flox/flox mice [29] with Cdh5(PAC)-Cre^ERT2^ mice [30]. All mice were of the C57BL/6 J genetic background. To induce genetic deletion of *Piezo1* gene, tamoxifen (Sigma Aldrich, #T5648) was prepared in corn oil (Sigma Aldrich, #C8267) at a concentration of 20 mg/mL and dissolved by shaking at 37°C. Mice at 5–6 weeks of age received intraperitoneal (i.p.) injection with 100 µL tamoxifen once a day for 5 consecutive days. Experiments were conducted at least 2 weeks after Cre induction. Tamoxifen treatment induced endothelial cell-specific deletion in Piezo1 EC-KO mice. The mice were anesthetized using 4 % isoflurane inhalation followed by maintaining doses of 2–3 % isoflurane during the imaging session (Doctor Oxygen Service, Inc., WI, USA). The hair from the abdomen area was removed using a trimmer and hair removing lotion.

### 2.3 Imaging protocol

In this proof-of-concept study, two mice per experimental group were utilized in each imaging session. The first mouse was Piezo1 flox/flox, while the second was Piezo1 EC-KO. Imaging was conducted at six different locations on the abdomen (Fig. 1b). To optimize the scanning process using the *s*LS-PAM system, the mice were placed in a supine position, ensuring direct contact of the abdomen with the water tank in the scanning area. A height-adjustable platform was employed to align the mouse with the system. Once the abdomen touched the water tank, the mouse was elevated until the abdominal skin reached the focal point of the focusing lens.

For imaging each specific region, the mouse was temporarily removed from the system and repositioned to align the center of the scan with the center of the region of interest. The focusing protocol was then repeated for each imaging session. This meticulous approach ensured accurate and optimal imaging of the targeted abdominal areas using the *s*LS-PAM system.

A second set of experiments was conducted using the same groups of animals, but this time, an intraperitoneal injection of 330 mg/kg BW 8Br-cAMP was administered. In this experiment, the right lower quadrant of the mouse was exposed to facilitate the insertion of the needle for intraperitoneal injection. This approach helped preserve the area of interest both before and after the injection, eliminating the necessity of removing the animal from the system, thus minimizing potential disruptions to the imaging process.

### 2.4 Ensuring data reliability

In some instances, after shaving and applying hair removal lotion, some residual hairs remained on the skin. In some cases, this led to high-intensity PA signals that made data acquisition from the vessels near the hair impossible to identify: these regions were excluded from analysis. Then, to maintain the accuracy and ensure a correct comparison, the decision was made to exclude from analysis the corresponding region from the other animal. By capturing data from identical regions for each animal, comparable vessels could be examined, thereby ensuring the consistency and reliability of the results despite the challenges encountered during the experiment.

An additional exclusion criterion was established for vessels that could not be separated (are aggregated) in the image. This situation occurs, for example, when veins and vessels are too close together, falling below the system's resolution. Such aggregations of vessels create a larger vessel that cannot be included in the analysis, as they represent a combination of two separate vessels. The intensity profiles of these cases are asymmetrical and do not fit well with a Gaussian distribution. Therefore, this characteristic was used to exclude them from the analysis.

## 3 Results and discussion

To assess the vessel diameters in the abdominal skin of control Piezo1 flox/flox and Piezo1 EC-KO mice, we used the PAM. Six regions were selected for each animal to comprehensively cover the mouse's abdominal skin area (see Fig. 1b). However, in the case of the last two pairs of animals, the experiment was conducted within only two regions. This modification was prompted by unforeseen issues with hair removal (see Section 2.4).

**Fig. 2:**
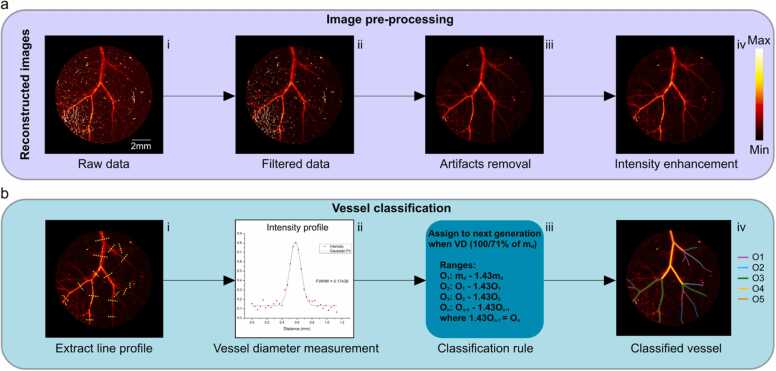
Image pre-processing and stepwise analysis of vessel morphology. (a) Image pre-processing starts with (i) the raw image and includes (ii) data filtering, (iii) artifact removal, and (iv) intensity enhancement. (b) Vessel classification includes (i) the extraction of the intensity profile for each segment of the imaged vessel tree; (ii) application of a gaussian fit to the vessel intensity profile for the diameter measurements; (iii) the classification of the vessel order based on the classification rule; (iv) further analysis of the classified segments. O_i_: ith -order of the vessel; m_d_: minimum diameter.

For precise vessel diameter measurements, we implemented an image pre-processing analysis (Fig. 2a). The initial step of this analysis involved filtering the raw data to eliminate background noise that could potentially interfere with the vessel diameter analysis (Fig. 2a.ii). We also removed artifacts arising from high-intensity signals, possibly caused by microbubbles in the ultrasound gel or hair (Fig. 2a.iii).

An intensity enhancement process was then applied to the images thereby improving vessel visibility (Fig. 2a.iv). To compare vessel diameters in different experimental groups, a vessel classification process was performed as follows (Fig. 2b). First, the diameter of each vessel was extracted from the intensity profile along the cross-section of the vessel (Fig. 2b.i). The full width at half maximum (FWHM) of the Gaussian fit to this profile was calculated as the vessel diameter (Fig. 2b.ii). This procedure was repeated ten times on nearby regions within each segment to enhance measurement accuracy. Next, we classified the vessels into different orders within the vessel tree. The classification process relied on the diameter of the smallest vessel within all those

collected for each group of animals. Since the smallest vessel diameters are in the same range when comparing animals inside the same group, it serves as a baseline measure for the classification. We did not choose the largest vessel as a reference because it was impractical. The largest vessel observed in one animal might represent a second-level vessel in another, creating a misleading basis for comparison.

Subsequently, vessels were assigned to the next order if their diameter was larger than 1.43 times the minimum diameter of the previous order (Fig. 2b. ii-iii). This number was derived from the assumption that effective blood flow within the vascular tree occurs only when the diameters of the two offspring vessels at branching bifurcation point are 71 % of the parent vessel [31], [32]. After this classification, the diameters of vessels from the same branching level were averaged [31], [33]. To ensure a statistically robust representation of the data, the same classification was applied to all images collected (see Methods).

**Fig. 3:**
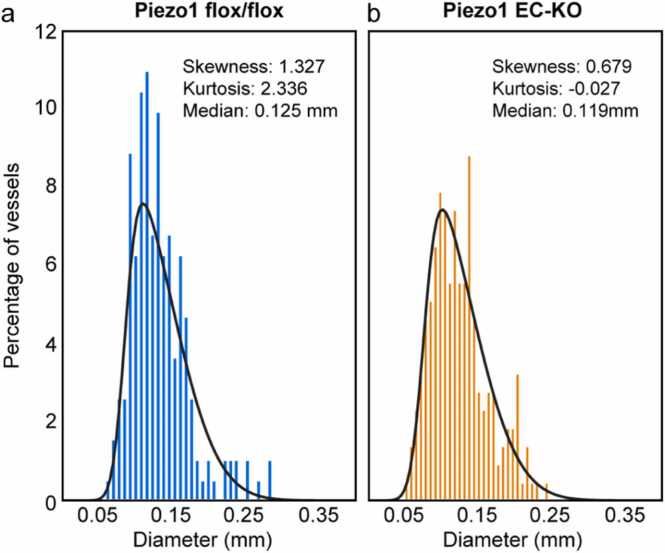
Vessel diameter distributions in the skin of Piezo1 flox/flox and Piezo1 EC-KO mice. The vessel diameters were extracted for each branch of the vessel tree, and a histogram was computed to compare the distribution of the vessel diameters in each experimental group. N = 5 animals per group.

To analyze the distribution of vessel diameters in each group of animals, we extracted the skewness and kurtosis values from the frequency histogram (Fig. 3). We found that the distributions from both groups were positively skewed; however, Piezo1 flox/flox mice exhibited slightly higher skewness compared to Piezo1 EC-KO mice. This indicates that while both groups predominantly feature small diameter vessels, the Piezo1 EC-KO group has a more concentrated distribution around smaller diameters. In contrast, the Piezo1 flox/flox group includes a few vessels with larger diameters, which skews the distribution toward larger sizes. Additionally, the Piezo1 EC-KO mice displayed lower kurtosis, suggesting that their data is less variable than in the control group.

In addition, we used the median as one of the parameters to describe the differences between the groups. The median was calculated as 0.125 mm [95 % CI: 0.117, 0.132] for the control Piezo1 flox/flox group and 0.119 mm [95 % CI: 0.113, 0.124] for the Piezo1 EC-KO group. The overlap in the confidence intervals of the two groups suggests there is no immediate evidence of a significant difference between them. However, the Mann-Whitney *U* test yielded a p-value of 0.016, indicating that the median diameter of the vessels from the Piezo1 EC-KO mice is statistically smaller than that of the Piezo1 flox/flox mice.

To further analyze and compare the diameter distributions between the groups, we classified the vessels to identify changes at each order level. Given the previous results, we cannot use the same criteria for classification in both groups, as one group exhibits smaller vessel diameters than the other. Therefore, using the same parameter for classification in both groups would lead to inaccuracies. For that reason, we used the smallest diameter identified for each group of mice as the reference for the order classification.

**Fig. 4:**
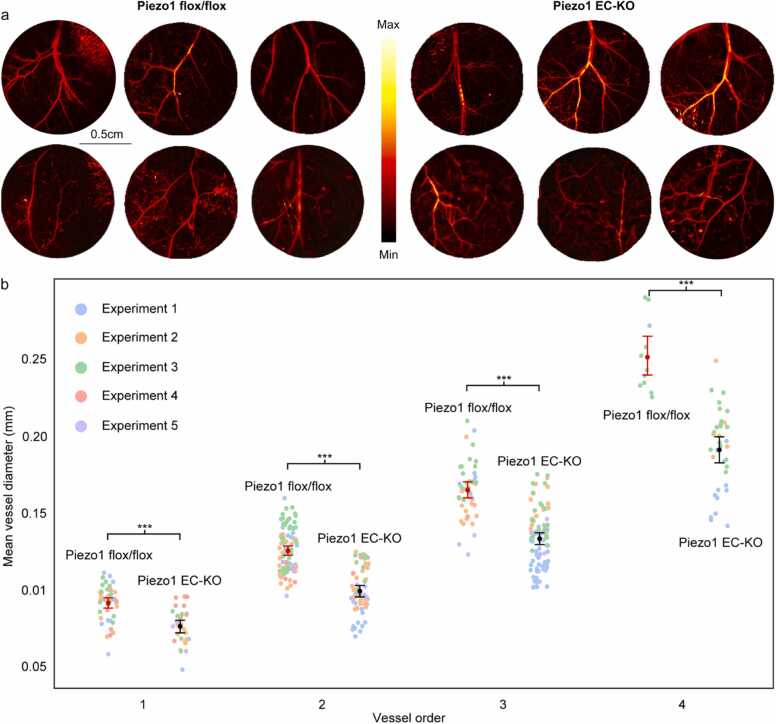
Comparison between diameter measurements for Piezo1 flox/flox and Piezo1 EC-KO mice. The comparison was made based on the mean diameter for each branching level in the vessels of the mouse skin, with 95 % confidence intervals. Statistics performed by unpaired one-tailed *t*-test: * , ** , and *** represent P < 0.05, P < 0.01, and P < 0.001, respectively. *ns* = not statistically significant. *N* = 5 animals per group.

As previously, the smallest group of vessels were considered as the highest-level vessel order within the tree, and we used the diameter of the smallest vessels to set up comparison between groups by vessel order (Fig. 4). The analyses of the vessel diameter per vessel order showed a significant difference between the vessels within corresponding order from the two experimental groups (Fig. 4). In addition to unpaired one-tailed t-test and Mann-Whitney *U* test, Cohen’s *d* was used to measure the effect size for the difference between the two groups. The results showed a significant difference between the two groups of animals at each order, Order 1: d = 1.31, 95 % CI [0.85, 1.94], Order 2: d = 1.77, 95 % CI [1.45, 2.15], Order 3: d = 1.72, 95 % CI [1.36, 2.17] and Order 4: d = 2.22, 95 % CI [1.66, 3.05].

**Fig. 5:**
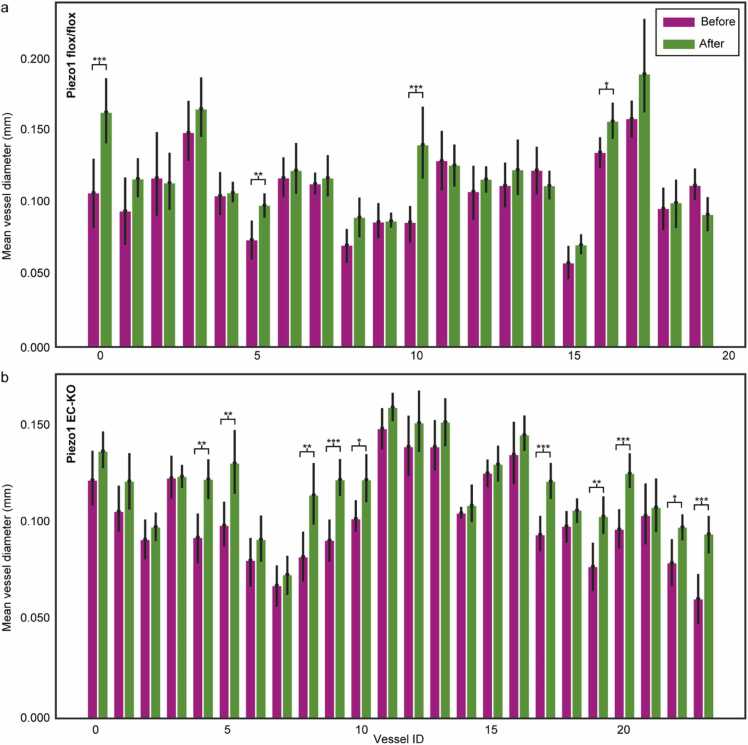
Vessel diameter before and after 8Br-cAMP injection. The comparison of the vessels for the flox/flox and EC-KO groups before and 10 minutes after the 8Br-cAMP injection was done by the diameter measurement as in the previous study, with 95 % confidence interval. For this comparison all the order of vessels were included for both groups and the individual change was evaluated. Statistics performed by unpaired one-tailed *t*-test, Mann-Whitney *U* test and Cohen’s d: * , ** , and *** represent P < 0.05 (d>0.2), P < 0.01(d>0.5), and P < 0.001 (d>0.8), respectively.

Next, we determined if intraperitoneal delivery of a membrane-permeant stable cAMP derivative, 8Br-cAMP [34], [35], can induce vessel vasodilation in Piezo1 EC-KO mice by inducing calcium release from IP_3_R2 stores [15]. In this set of the experiments, all mice were imaged before and 10 minutes after an intraperitoneal injection of 8Br-cAMP (Fig. 5). Following the injection of 8Br-cAMP, there was an overall increase in the diameters of vessels in both experimental groups, indicating vasodilation. In the control group, only 4 out of 20 vessels showed a significant increase in diameter. In contrast, the Piezo1 EC-KO group had a much higher number, with 10 out of 24 vessels exhibiting this change. This suggests that the injection caused more pronounced effects in

the Piezo1 EC-KO group compared to the control group. The statistical analysis was performed using an unpaired one-tailed *t*-test, Mann-Whitney *U* test, and Cohen’s d to measure the effect size for the difference between the mean diameter of the vessel before and after 8Br-cAMP injection.

**Fig. 6:**
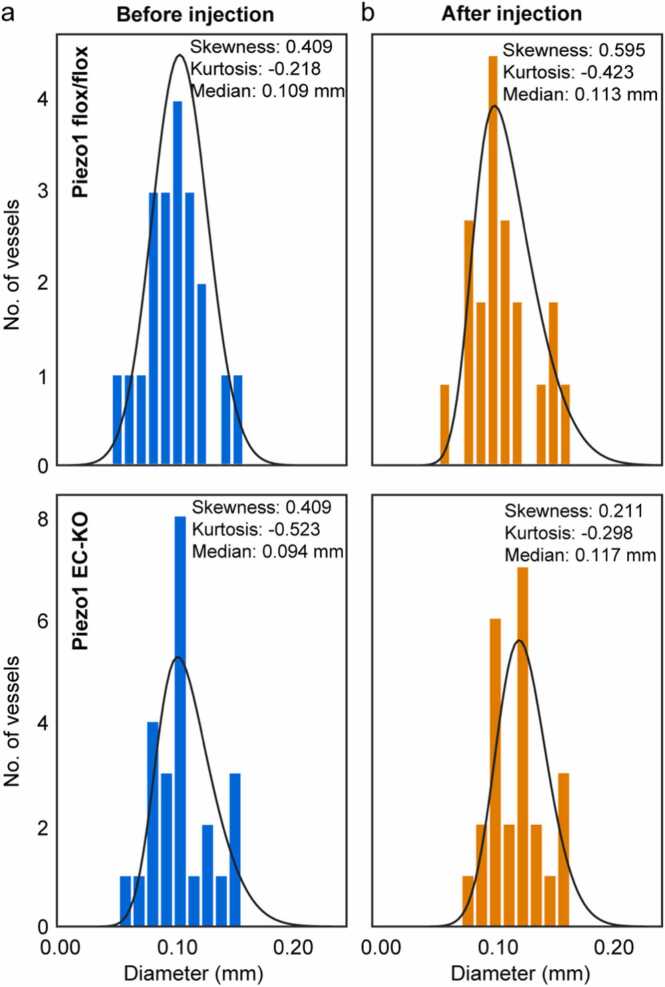
Vessel diameter distributions in Piezo1 flox/flox and Piezo1ΔEC mice before and after 8Br-cAMP injection. Vasodilated Piezo1 EC-KO mice responses to 8Br-cAMP are greater than vasodilated Piezo1 flox/flox mice responses.

A histogram distribution of the vessel diameter was calculated for both groups of animals before and after 8Br-cAMP injection (Fig. 6). For the Piezo1 flox/flox vessels, the skewness of the distribution increased after the injection, suggesting that the distribution of the vessels became more asymmetric shifting towards larger values. This can be interpreted to show that the larger vessels dilated more than smaller vessels. The median of vessel diameter also increased after injection. Still, this difference was not statistically significant based on the Mann-Whitney *U* test (*p*-value, 0.161), one-tailed *t*-test (p-value, 0.108), and effect size Cohen’s d test (d-value, 0.39).

In Piezo1 EC-KO mice, we also observed increase in the median vessel diameter after 8Br-cAMP injection (Fig. 6). In addition, this increment was higher than in the control group suggesting a greater effect of 8Br-cAMP on vessel dilation in the experimental group. In fact, the observed changes in the Piezo1 EC-KO group were highly significant (*p*-value, 0.007, d-value, 0.715). Furthermore, the skewness decreased after the injection of 8Br-cAMP in Piezo1 EC-KO group meaning the smaller vessels showed a larger increase toward the median. This could mean that vessel distribution is more balanced after the injection. Cumulatively, our results suggest that the injection of 8Br-cAMP had a differential effect on vessel diameter between the two groups tested.

**Fig. 7:**
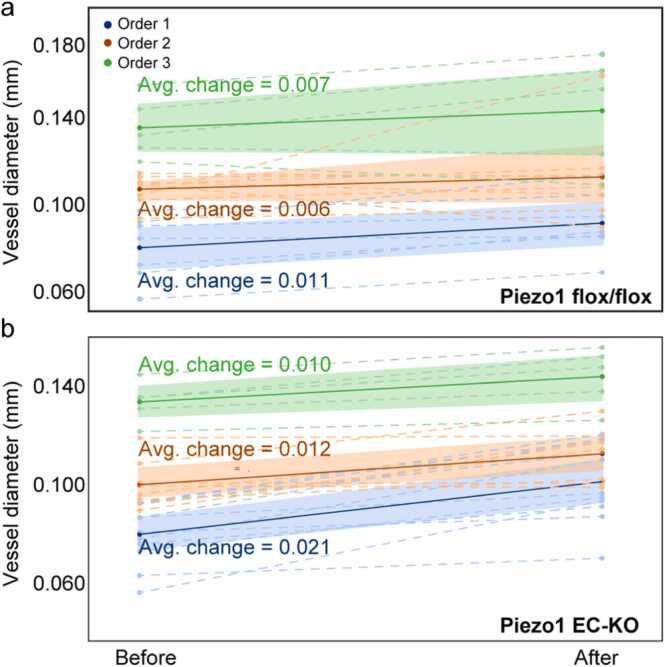
Changes in corresponding vessel diameters before and after 8Br-cAMP injection. The vessels were classified to each order based on the vessel size acquired before the injection, then the diameter sizes were plotted for both cases. An average change is shown (in mm) to compare the effect of the injection in the two experimental groups for each order of vessels, with 95 % confidence intervals. Smaller vessels of Piezo1 EC-KO mice showed a greater increase in diameter as a result of vasodilation than Piezo1 flox/flox mice.

Moreover, using the classification methods described here, we also evaluated wherever the injection affects all vessels in the same manner. The vessels were classified based on their baseline diameters (before the injection), and then the diameters of the same vessels were plotted before and after the injection for the matched groups (Fig. 7). We observed that the smaller vessels experienced greater changes in the diameter after 8Br-cAMP injection in both experimental groups (Fig. 7). Besides, from all the vessel orders, the effect of 8Br-cAMP was greater in smaller vessels of Piezo1 EC-KO mice (0.021 mm [95 % CI: 0.019, 0.023]) compared with 0.011 mm [95 % CI: 0.006, 0.012] in Piezo1 flox/flox mice. Additionally, the confidence intervals indicate that after the injection, the changes in vessel structure for the Piezo1 EC-KO group exhibit less variability, suggesting a more pronounced increase in vessel size compared to the changes for the Piezo1 flox/flox group.

Cumulatively, these results indicate that hypertension in mice associated with endothelial Piezo1 deficiency is not solely due to impaired Ca^2+^ influx through the channel but also partially due to impaired Ca^2+^ realse from sAC-sensitive intraceullular compartments. This is demonstrated by the phnotypic rescue observed upon injecting a stable cAMP analog, which bypasses Piezo1 activation and Ca^2+^ influx, potentiates calcium release from IP_3_R2 stores [15] and promotes vasodilation of resistance arteries.

The methodology developed in this study serves as a valuable tool for monitoring the morphological changes in the skin vessels associated with the inactivation of the mechanosensitive channel Piezo1 in endothelial cells (Piezo EC-KO [36]). These alterations significantly impact vascular function. When the Piezo1 channel is deleted, endothelial cells lose their ability to effectively sense and respond to changes in blood flow [9], [15], [36], [37]. This methodology effectively distinguishes between populations of mice with a deficiency in the Piezo1 mechanosensitive channel and demonstrates the effects of pharmacological agents on blood vessel vasodilation.

The method categorizes vessels based on their diameter, enabling a comparative analysis of vascular morphological changes within the same group of vessels across different animals. Moreover, this methodology has the potential to be applied in studies of vascular mechanodynamics, contributing to the development of treatments and advancing research in the field of hypertensive disorders. We demonstrated that this method serves as an insightful tool for assessing the effects of pharmacological interventions on vascular tone. Our findings indicate that direct activation of sAC can effectively induce vasodilation even in the absence of functional Piezo1channel. This suggests a potential therapeutic approach for conditions such as hypertension, where blood vessels fail to respond appropriately to changes in blood pressure. In our methodology, we measured the impact of drug treatments on blood vessels in Piezo1-deficient mice in real-time. Upon administering the cAMP analog 8Br-cAMP to mice, we observed a significant increase in the diameter of constricted vessels in Piezo1 EC-KO mice compared to control mice. This indicates that cAMP pathways could improve blood vessel function even without Piezo1, providing a possible solution for conditions that involve altered responses to blood pressure and flow. Particularly, this study is the first to demonstrate that cAMP analogs may be effective in managing chronic or sudden hypertension by rapidly lowering blood pressure and reducing the risk of tissue damage and stroke. Our research on mice with chronic high blood pressure [9] showed that cAMP analog8Br-cAMP can widen blood vessels and improve tissue perfusion within just a few minutes. This rapid response makes them a promising option for treating acute high blood pressure crises.

## 4 Conclusion

We demonstrated the utility of sLS-PAM in analyzing the responses of resistance arteries to both mechanical stress and pharmacological treatment. The observation that Piezo1 EC-KO mice exhibited smaller vessel diameters highlights the crucial role of Piezo1-mediated mechanotransduction in regulating vascular endothelial cell responses to subtle changes in blood pressure. This supports the overall view on Piezo1’s function as a mechanosensitive ion channel essential for maintaining proper vascular tone and adapting to hemodynamic changes.

The analysis of vessel diameter distributions provided valuable insights into the structural vascular phenotype associated with Piezo1 deletion. Notably, the difference in the median diameter size toward smaller vessels in Piezo1 EC-KO mice compared to control Piezo1 flox/flox animals indicates a pronounced shift in vessel network architecture. This finding aligns with the chronic hypertension observed in Piezo1 EC-KO mice [9], suggests that Piezo1 deletion leads to maladaptive changes in vascular structure and impaired network remodeling. These structural alterations likely exacerbate the inability of resistance arteries to respond appropriately to fluctuations in blood flow, further contributing to elevated blood pressure as well as poor perfusion of tissue.

Additionally, we demonstrated that bypassing Piezo1 function through direct activation of sAC is an effective strategy for inducing vasodilation in vessels lacking adaptive responses to blood flow. This highlights a potential therapeutic avenue for targeting Piezo1-deficient or dysfunctional endothelial systems. Using the sLS-PAM system, we were able to measure real-time, in vivo effects of pharmacological treatment on vessel structure in the context of Piezo1 deletion. The application of the cAMP analog 8Br-cAMP led to a significantly greater increase in the diameters of smaller, vasoconstricted vessels in Piezo1 EC-KO mice compared to control Piezo1 flox/flox mice. This finding underscores the potential for cAMP-mediated pathways to restore vascular function even in the absence of Piezo1, offering a promising approach for conditions characterized by mechanotransduction deficits.

Although stable cAMP analogs, such as 8-chloro-cAMP, have been shown to exhibit anti-tumor activity [38], [39] and have even been tested in Phase 1 clinical studies [40], [41], their potential application for the treatment of hypertension has not been previously explored. This study is the first to demonstrate the potential use of cAMP analogs for managing acute hypertensive events, with the goal of rapidly reducing blood pressure and mitigating the risk of tissue ischemia and stroke.

Currently, the first-line treatments for hypertensive emergencies typically involve the intravenous administration of beta-blockers, calcium channel blockers, or other rapid-acting antihypertensive agents [42]. While these medications are effective, they often require hours to achieve their full therapeutic effect, a timeframe that may not be sufficient to prevent complications in critical situations. By contrast, our data in chronically hypertensive mice reveal that cAMP analogs, such as 8Br-cAMP, can induce vasodilation and reduce blood pressure within minutes. This rapid onset of action highlights their potential as a complementary or alternative therapy for acute hypertensive crises, where immediate reduction of vascular resistance is paramount.

The observed efficacy of cAMP analogs in Piezo1-deficient mice further underscores their therapeutic promise. Piezo1 deletion in endothelial cells compromises mechanotransduction, leading to chronic hypertension and impaired vascular responsiveness. In our study, cAMP analogs bypassed the need for Piezo1 function, directly activating downstream pathways that promote endothelial nitric oxide synthase (eNOS) activity and vasodilation. This suggests that cAMP analogs could be particularly beneficial in conditions where mechanotransduction is dysfunctional or impaired, such as in certain forms of vascular disease or genetic disorders affecting Piezo1 signaling.

The promising preclinical findings reported here warrant further investigation into the safety, pharmacokinetics, and efficacy of cAMP analogs in both acute and chronic hypertensive models. Future studies should also explore their combination with existing antihypertensive agents to determine whether synergistic effects can be achieved. Additionally, the role of cAMP signaling in other vascular pathologies, such as endothelial dysfunction or atherosclerosis, could provide new avenues for therapeutic development.

Furthermore, the results of this study establish sLS-PAM as a powerful tool for assessing both structural and functional changes in resistance arteries, with potential for seamless translation into clinical trials to evaluate the effects of novel antihypertensive agents. They also reinforce the importance of Piezo1 in vascular homeostasis while identifying alternative mechanisms, such as sAC activation, that may compensate for Piezo1 dysfunction in pathological states. Future studies should further investigate the interplay between Piezo1 signaling and downstream pathways, such as sAC-cAMP signaling, to uncover additional therapeutic opportunities for managing vascular dysfunction and hypertension.

## Funding

This work was supported by the 10.13039/100000002National Institutes of Health; R01EB027769, R01EB028661 (Kamran Avanaki), R01HL045638 (Yulia Komarova).

## CRediT authorship contribution statement

**Zafar Mohsin:** Software, Methodology, Data curation. **Santana Nunez Dianicha:** Writing – review & editing, Methodology, Investigation, Data curation, Conceptualization. **Benavides**-**Lara Juliana:** Writing – review & editing, Writing – original draft, Visualization, Software, Methodology, Investigation, Formal analysis, Data curation. **Avanaki Kamran:** Writing – review & editing, Supervision, Project administration, Funding acquisition, Formal analysis, Conceptualization. **Komarova Yulia A:** Writing – review & editing, Writing – original draft, Supervision, Project administration, Funding acquisition, Formal analysis, Conceptualization. **Zhao Shuangping:** Project administration, Methodology, Investigation, Data curation. **Garcia Campos Janette:** Writing – original draft.

## Declaration of Competing Interest

The authors declare the following financial interests/personal relationships which may be considered as potential competing interests: Kamran Avanaki and Yulia Komarova reports financial support was provided by National Institutes of Health. If there are other authors, they declare that they have no known competing financial interests or personal relationships that could have appeared to influence the work reported in this paper.

## Data Availability

Data will be made available on request.
